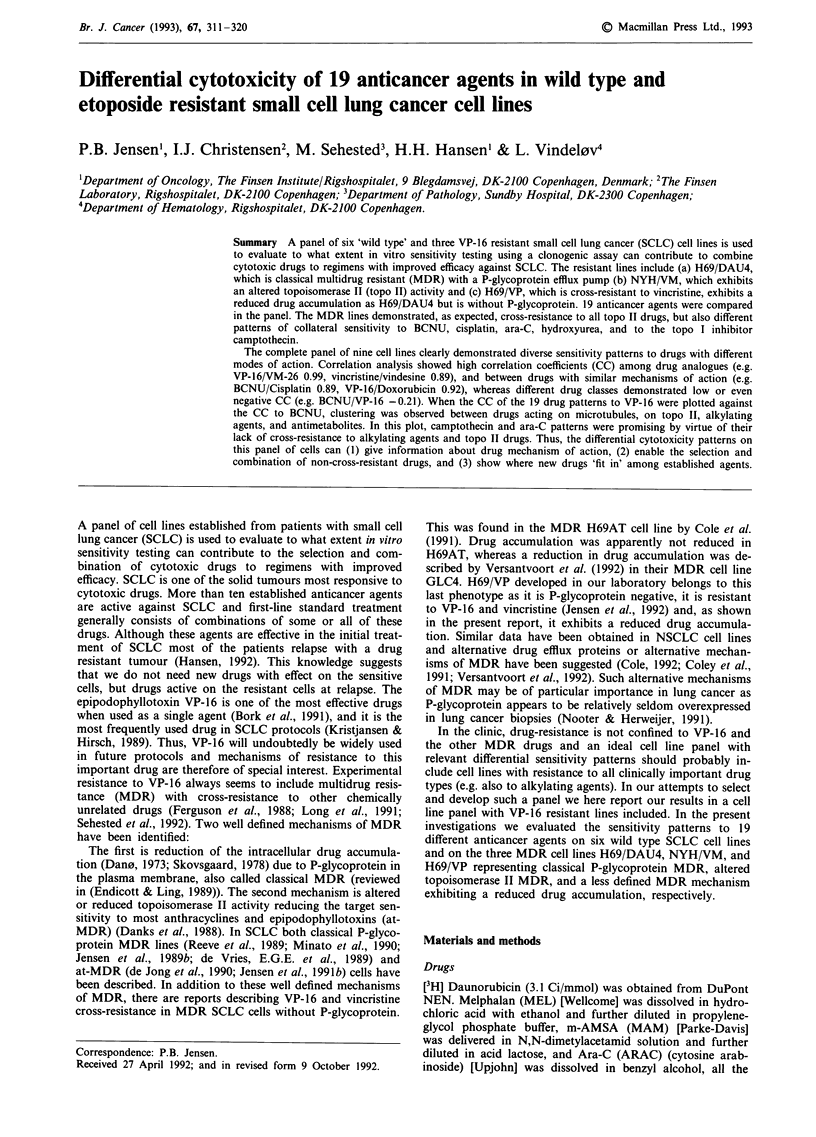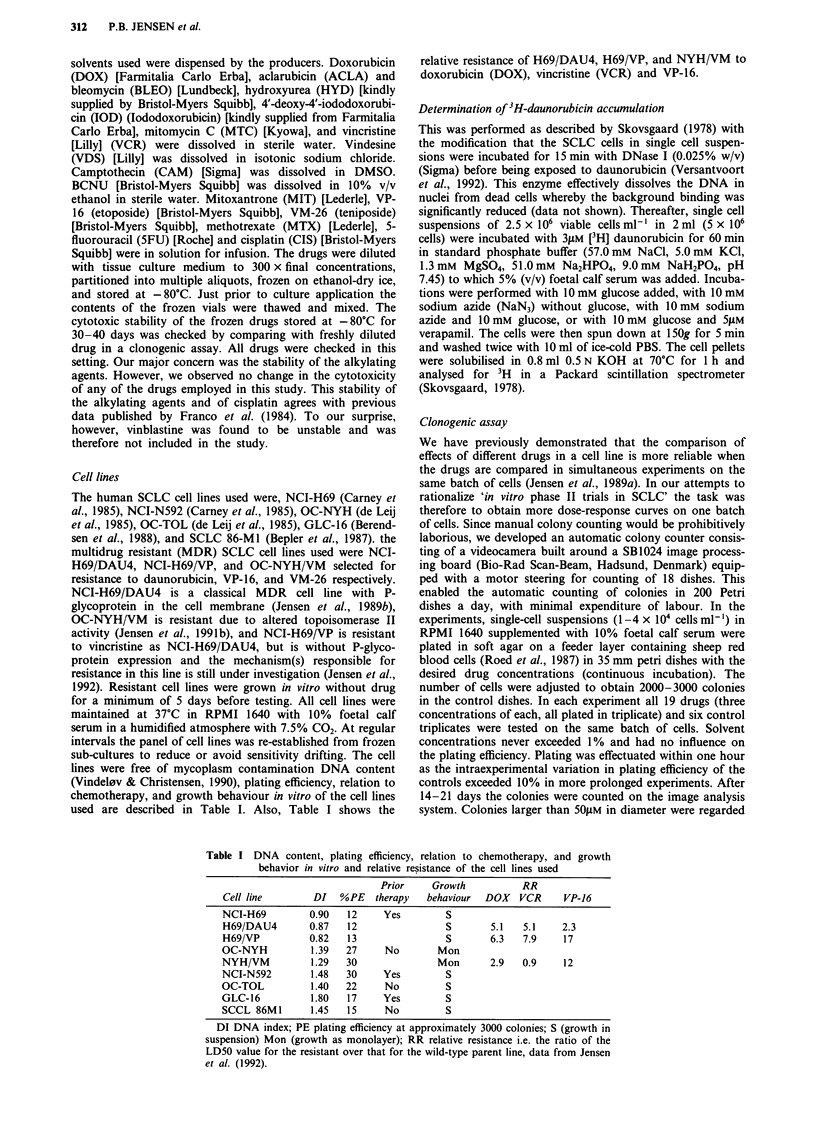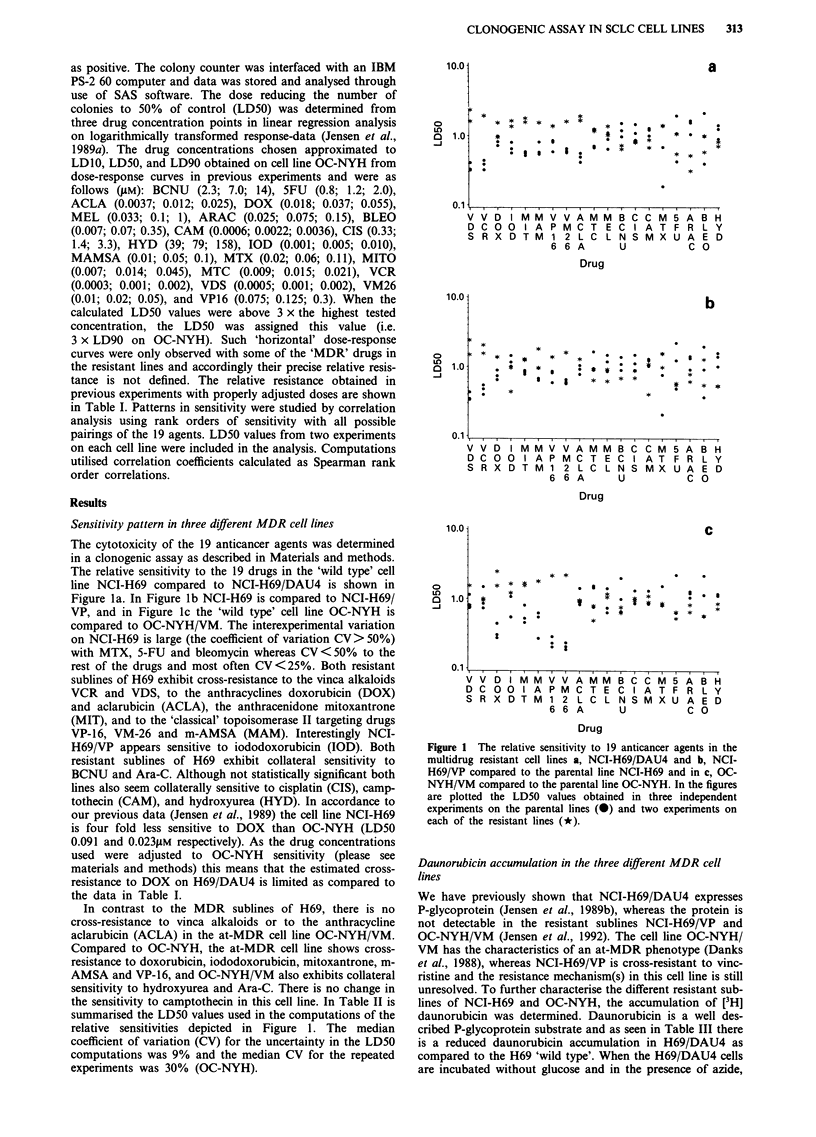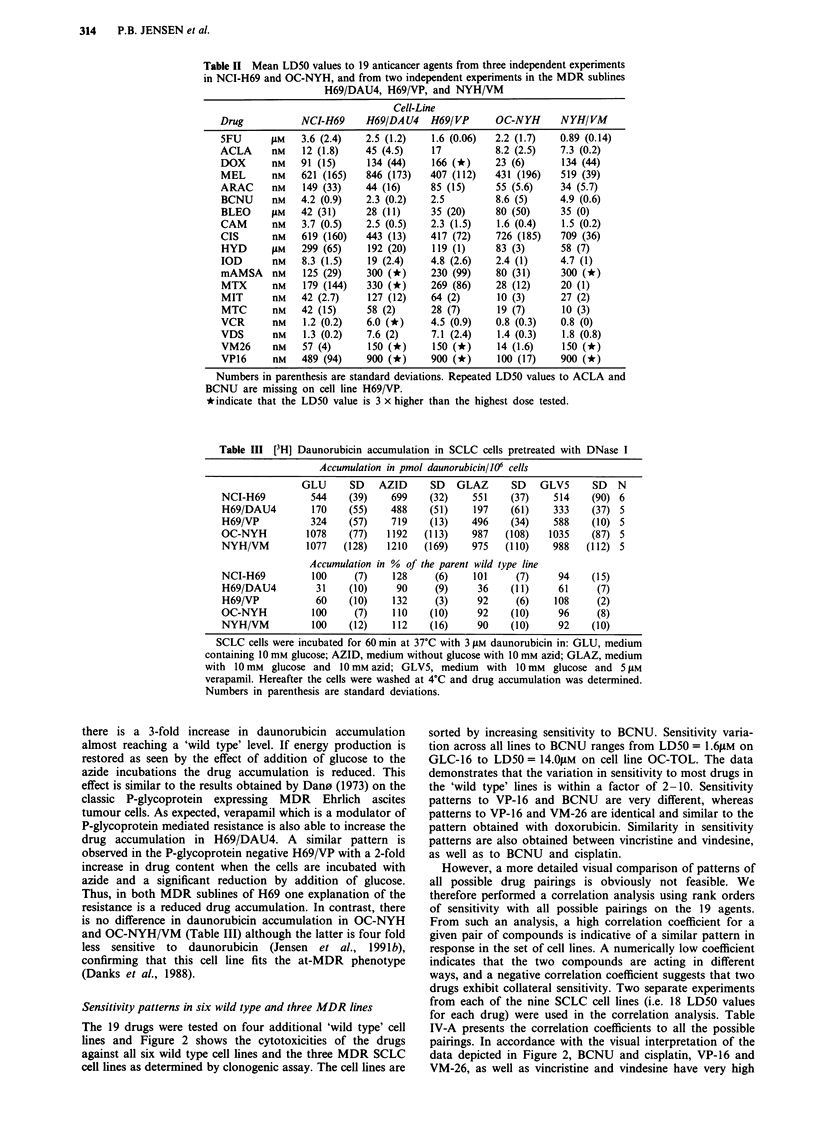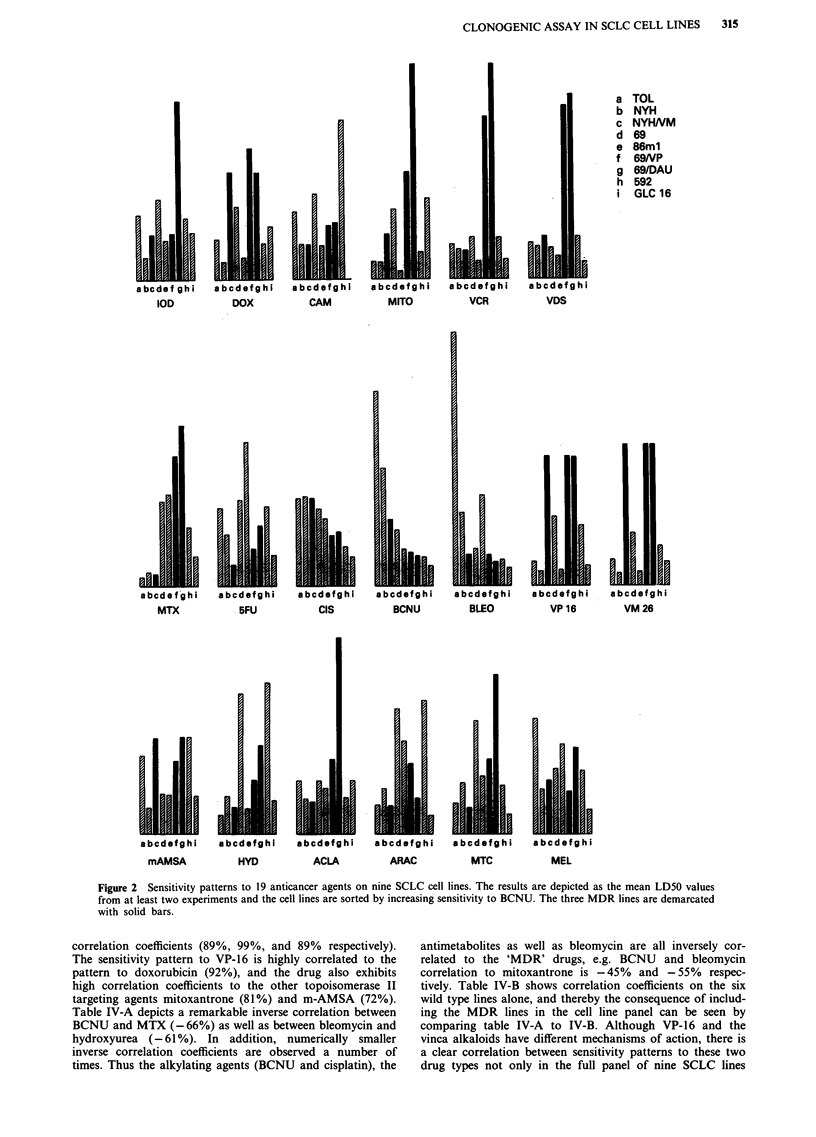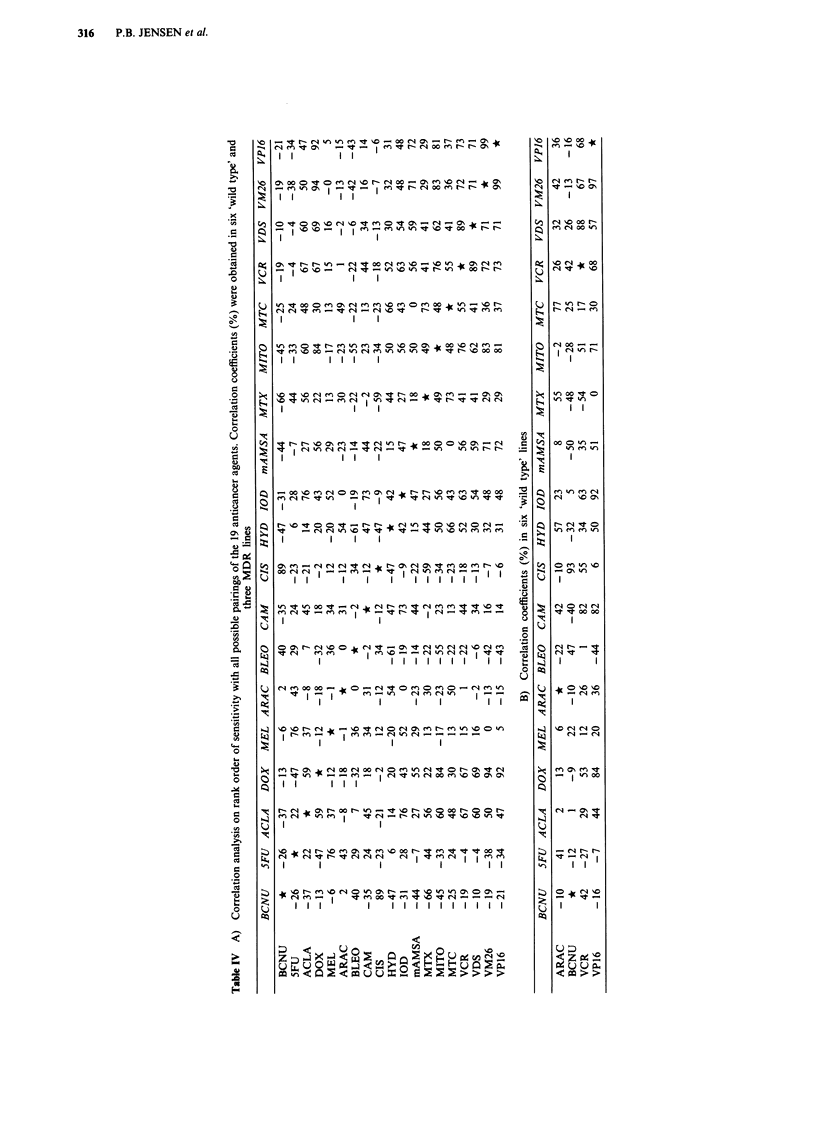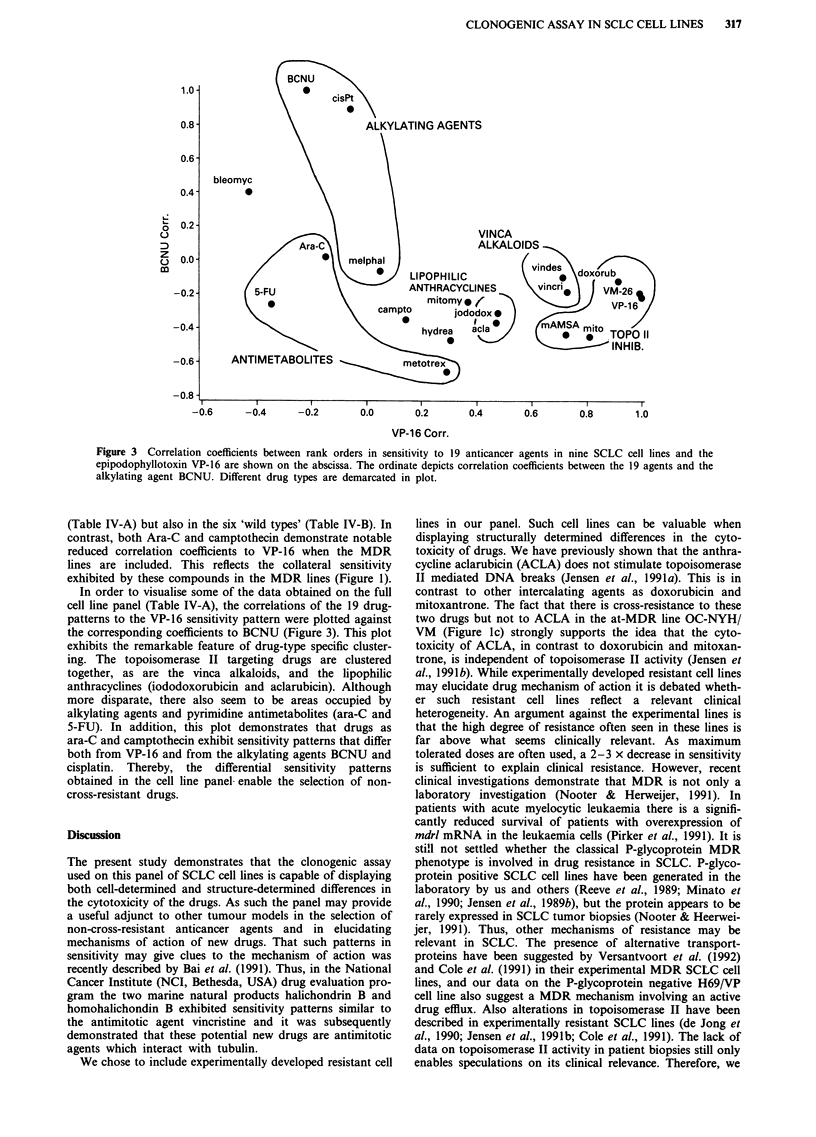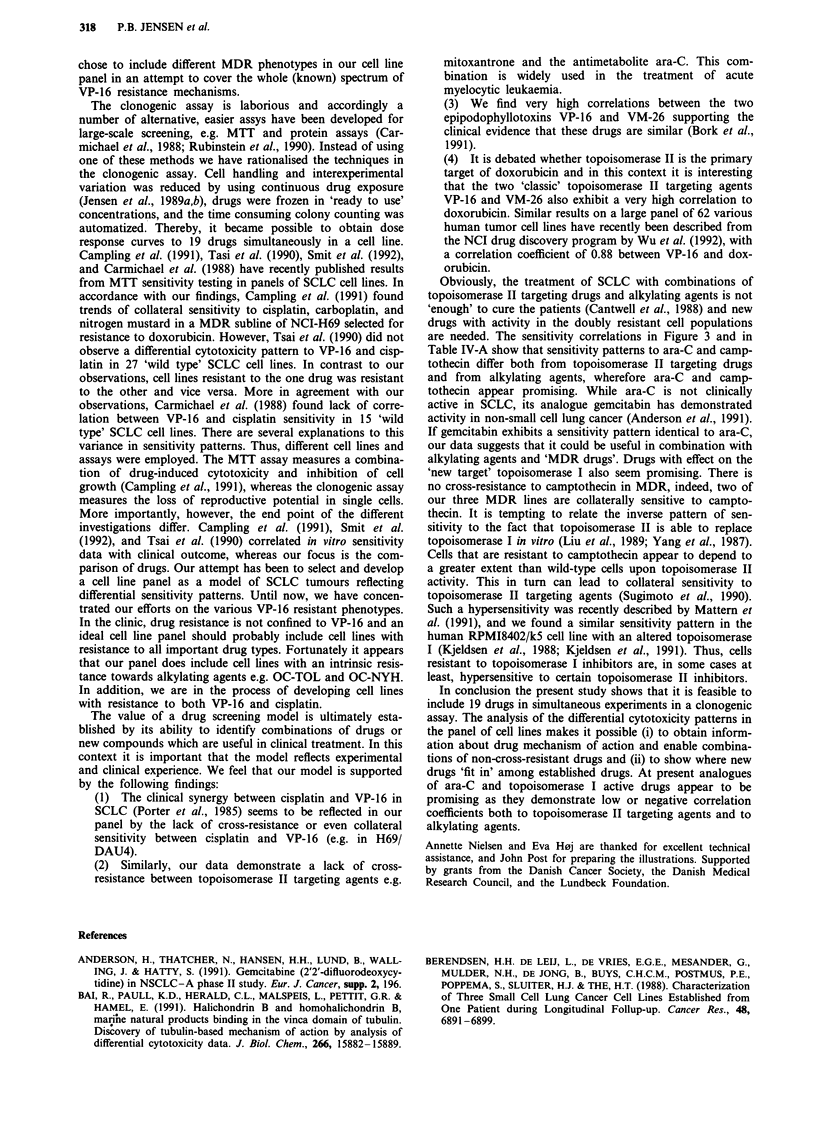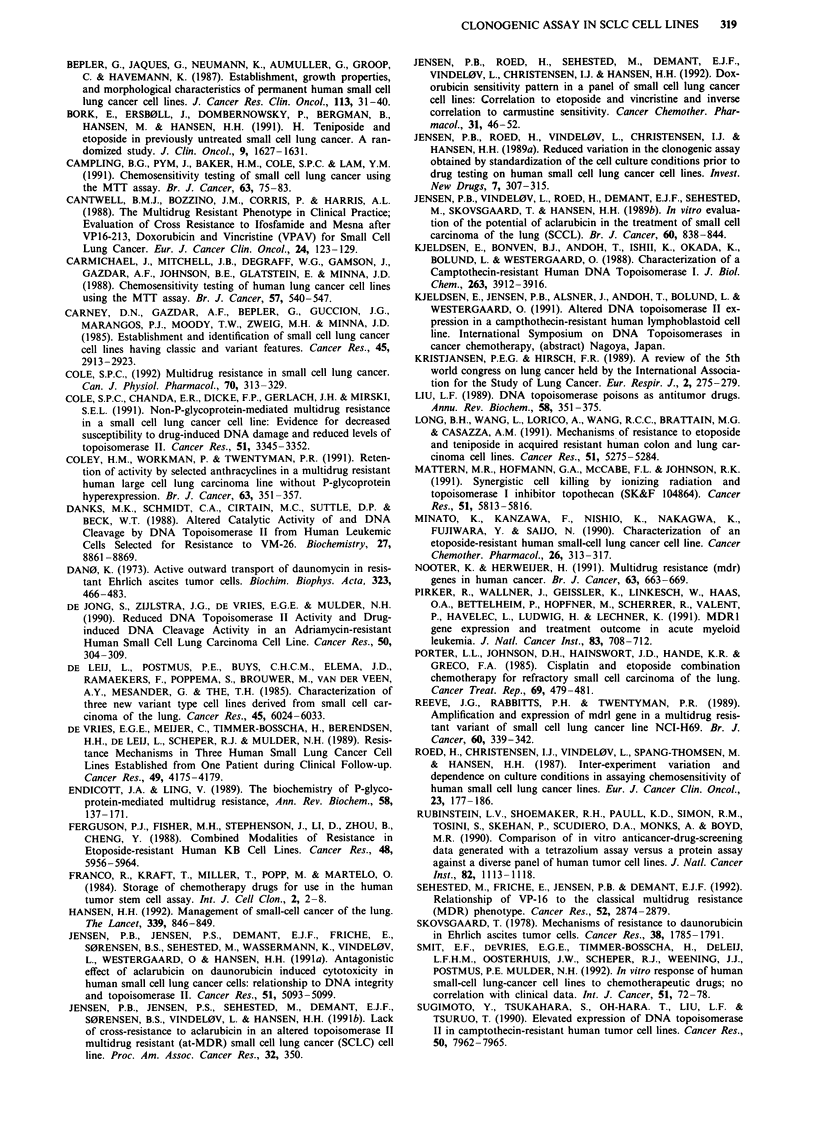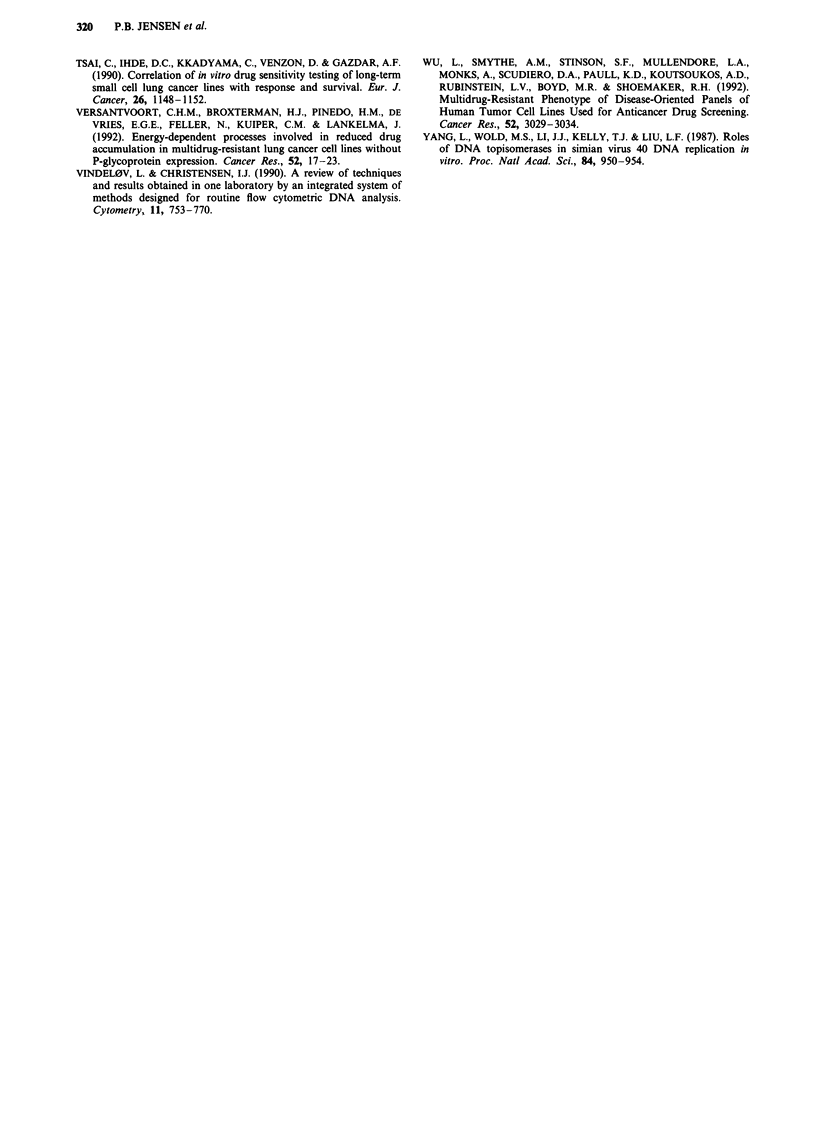# Differential cytotoxicity of 19 anticancer agents in wild type and etoposide resistant small cell lung cancer cell lines.

**DOI:** 10.1038/bjc.1993.58

**Published:** 1993-02

**Authors:** P. B. Jensen, I. J. Christensen, M. Sehested, H. H. Hansen, L. Vindeløv

**Affiliations:** Department of Oncology, Finsen Institute/Rigshospitalet, Copenhagen, Denmark.

## Abstract

A panel of six 'wild type' and three VP-16 resistant small cell lung cancer (SCLC) cell lines is used to evaluate to what extent in vitro sensitivity testing using a clonogenic assay can contribute to combine cytotoxic drugs to regimens with improved efficacy against SCLC. The resistant lines include (a) H69/DAU4, which is classical multidrug resistant (MDR) with a P-glycoprotein efflux pump (b) NYH/VM, which exhibits an altered topoisomerase II (topo II) activity and (c) H69/VP, which is cross-resistant to vincristine, exhibits a reduced drug accumulation as H69/DAU4 but is without P-glycoprotein. 19 anticancer agents were compared in the panel. The MDR lines demonstrated, as expected, cross-resistance to all topo II drugs, but also different patterns of collateral sensitivity to BCNU, cisplatin, ara-C, hydroxyurea, and to the topo I inhibitor camptothecin. The complete panel of nine cell lines clearly demonstrated diverse sensitivity patterns to drugs with different modes of action. Correlation analysis showed high correlation coefficients (CC) among drug analogues (e.g. VP-16/VM-26 0.99, vincristine/vindesine 0.89), and between drugs with similar mechanisms of action (e.g. BCNU/Cisplatin 0.89, VP-16/Doxorubicin 0.92), whereas different drug classes demonstrated low or even negative CC (e.g. BCNU/VP-16 -0.21). When the CC of the 19 drug patterns to VP-16 were plotted against the CC to BCNU, clustering was observed between drugs acting on microtubules, on topo II, alkylating agents, and antimetabolites. In this plot, camptothecin and ara-C patterns were promising by virtue of their lack of cross-resistance to alkylating agents and topo II drugs. Thus, the differential cytotoxicity patterns on this panel of cells can (1) give information about drug mechanism of action, (2) enable the selection and combination of non-cross-resistant drugs, and (3) show where new drugs 'fit in' among established agents.


Br. J. Cancer (1993), 67, 311–320 © Macmillan Press Ltd., 1993

### Differential cytotoxicity of 19 anticancer agents in wild type and etoposide resistant small cell lung cancer cell lines

P.B. Jensen[1], I.J. Christensen[2], M. Sehested[3], H.H. Hansen[1] & L. Vindeløv[4]

[1]*Department of Oncology, The Finsen Institute/Rigshospitalet, 9 Blegdamsvej, DK-2100 Copenhagen, Denmark;* [2]*The Finsen Laboratory, Rigshospitalet, DK-2100 Copenhagen;* [3]*Department of Pathology, Sundby Hospital, DK-2300 Copenhagen;* [4]*Department of Hematology, Rigshospitalet, DK-2100 Copenhagen.*

**Summary** A panel of six 'wild type' and three VP-16 resistant small cell lung cancer (SCLC) cell lines is used to evaluate to what extent in vitro sensitivity testing using a clonogenic assay can contribute to combine cytotoxic drugs to regimens with improved efficacy against SCLC. The resistant lines include (a) H69/DAU4, which is classical multidrug resistant (MDR) with a P-glycoprotein efflux pump (b) NYH/VM, which exhibits an altered topoisomerase II (topo II) activity and (c) H69/VP, which is cross-resistant to vincristine, exhibits a reduced drug accumulation as H69/DAU4 but is without P-glycoprotein. 19 anticancer agents were compared in the panel. The MDR lines demonstrated, as expected, cross-resistance to all topo II drugs, but also different patterns of collateral sensitivity to BCNU, cisplatin, ara-C, hydroxyurea, and to the topo I inhibitor camptothecin.

The complete panel of nine cell lines clearly demonstrated diverse sensitivity patterns to drugs with different modes of action. Correlation analysis showed high correlation coefficients (CC) among drug analogues (e.g. VP-16/VM-26 0.99, vincristine/vindesine 0.89), and between drugs with similar mechanisms of action (e.g. BCNU/Cisplatin 0.89, VP-16/Doxorubicin 0.92), whereas different drug classes demonstrated low or even negative CC (e.g. BCNU/VP-16 −0.21). When the CC of the 19 drug patterns to VP-16 were plotted against the CC to BCNU, clustering was observed between drugs acting on microtubules, on topo II, alkylating agents, and antimetabolites. In this plot, camptothecin and ara-C patterns were promising by virtue of their lack of cross-resistance to alkylating agents and topo II drugs. Thus, the differential cytotoxicity patterns on this panel of cells can (1) give information about drug mechanism of action, (2) enable the selection and combination of non-cross-resistant drugs, and (3) show where new drugs 'fit in' among established agents.

A panel of cell lines established from patients with small cell lung cancer (SCLC) is used to evaluate to what extent *in vitro* sensitivity testing can contribute to the selection and combination of cytotoxic drugs to regimens with improved efficacy. SCLC is one of the solid tumours most responsive to cytotoxic drugs. More than ten established anticancer agents are active against SCLC and first-line standard treatment generally consists of combinations of some or all of these drugs. Although these agents are effective in the initial treatment of SCLC most of the patients relapse with a drug resistant tumour (Hansen, 1992). This knowledge suggests that we do not need new drugs with effect on the sensitive cells, but drugs active on the resistant cells at relapse. The epipodophyllotoxin VP-16 is one of the most effective drugs when used as a single agent (Bork *et al.*, 1991), and it is the most frequently used drug in SCLC protocols (Kristjansen & Hirsch, 1989). Thus, VP-16 will undoubtedly be widely used in future protocols and mechanisms of resistance to this important drug are therefore of special interest. Experimental resistance to VP-16 always seems to include multidrug resistance (MDR) with cross-resistance to other chemically unrelated drugs (Ferguson *et al.*, 1988; Long *et al.*, 1991; Sehested *et al.*, 1992). Two well defined mechanisms of MDR have been identified:

The first is reduction of the intracellular drug accumulation (Danø, 1973; Skovsgaard, 1978) due to P-glycoprotein in the plasma membrane, also called classical MDR (reviewed in (Endicott & Ling, 1989)). The second mechanism is altered or reduced topoisomerase II activity reducing the target sensitivity to most anthracyclines and epipodophyllotoxins (at-MDR) (Danks *et al.*, 1988). In SCLC both classical P-glycoprotein MDR lines (Reeve *et al.*, 1989; Minato *et al.*, 1990; Jensen *et al.*, 1989*b*; de Vries, E.G.E. *et al.*, 1989) and at-MDR (de Jong *et al.*, 1990; Jensen *et al.*, 1991*b*) cells have been described. In addition to these well defined mechanisms of MDR, there are reports describing VP-16 and vincristine cross-resistance in MDR SCLC cells without P-glycoprotein. This was found in the MDR H69AT cell line by Cole *et al.* (1991). Drug accumulation was apparently not reduced in H69AT, whereas a reduction in drug accumulation was described by Versantvoort *et al.* (1992) in their MDR cell line GLC4. H69/VP developed in our laboratory belongs to this last phenotype as it is P-glycoprotein negative, it is resistant to VP-16 and vincristine (Jensen *et al.*, 1992) and, as shown in the present report, it exhibits a reduced drug accumulation. Similar data have been obtained in NSCLC cell lines and alternative drug efflux proteins or alternative mechanisms of MDR have been suggested (Cole, 1992; Coley *et al.*, 1991; Versantvoort *et al.*, 1992). Such alternative mechanisms of MDR may be of particular importance in lung cancer as P-glycoprotein appears to be relatively seldom overexpressed in lung cancer biopsies (Nooter & Herweijer, 1991).

In the clinic, drug-resistance is not confined to VP-16 and the other MDR drugs and an ideal cell line panel with relevant differential sensitivity patterns should probably include cell lines with resistance to all clinically important drug types (e.g. also to alkylating agents). In our attempts to select and develop such a panel we here report our results in a cell line panel with VP-16 resistant lines included. In the present investigations we evaluated the sensitivity patterns to 19 different anticancer agents on six wild type SCLC cell lines and on the three MDR cell lines H69/DAU4, NYH/VM, and H69/VP representing classical P-glycoprotein MDR, altered topoisomerase II MDR, and a less defined MDR mechanism exhibiting a reduced drug accumulation, respectively.

### Materials and methods

### *Drugs*

[$^3$H] Daunorubicin (3.1 Ci/mmol) was obtained from DuPont NEN. Melphalan (MEL) [Wellcome] was dissolved in hydrochloric acid with ethanol and further diluted in propylene-glycol phosphate buffer, m-AMSA (MAM) [Parke-Davis] was delivered in N,N-dimetylacetamid solution and further diluted in acid lactose, and Ara-C (ARAC) (cytosine arabinoside) [Upjohn] was dissolved in benzyl alcohol, all the

Correspondence: P.B. Jensen.
Received 27 April 1992; and in revised form 9 October 1992.

solvents used were dispensed by the producers. Doxorubicin (DOX) [Farmitalia Carlo Erba], aclarubicin (ACLA) and bleomycin (BLEO) [Lundbeck], hydroxyurea (HYD) [kindly supplied by Bristol-Myers Squibb], 4'-deoxy-4'-iododoxorubicin (IOD) (Iododoxorubicin) [kindly supplied from Farmitalia Carlo Erba], mitomycin C (MTC) [Kyowa], and vincristine [Lilly] (VCR) were dissolved in sterile water. Vindesine (VDS) [Lilly] was dissolved in isotonic sodium chloride. Camptothecin (CAM) [Sigma] was dissolved in DMSO. BCNU [Bristol-Myers Squibb] was dissolved in 10% v/v ethanol in sterile water. Mitoxantrone (MIT) [Lederle], VP-16 (etoposide) [Bristol-Myers Squibb], VM-26 (teniposide) [Bristol-Myers Squibb], methotrexate (MTX) [Lederle], 5-fluorouracil (5FU) [Roche] and cisplatin (CIS) [Bristol-Myers Squibb] were in solution for infusion. The drugs were diluted with tissue culture medium to 300 × final concentrations, partitioned into multiple aliquots, frozen on ethanol-dry ice, and stored at −80°C. Just prior to culture application the contents of the frozen vials were thawed and mixed. The cytotoxic stability of the frozen drugs stored at −80°C for 30–40 days was checked by comparing with freshly diluted drug in a clonogenic assay. All drugs were checked in this setting. Our major concern was the stability of the alkylating agents. However, we observed no change in the cytotoxicity of any of the drugs employed in this study. This stability of the alkylating agents and of cisplatin agrees with previous data published by Franco et al. (1984). To our surprise, however, vinblastine was found to be unstable and was therefore not included in the study.

### Cell lines

The human SCLC cell lines used were, NCI-H69 (Carney et al., 1985), NCI-N592 (Carney et al., 1985), OC-NYH (de Leij et al., 1985), OC-TOL (de Leij et al., 1985), GLC-16 (Berendsen et al., 1988), and SCLC 86-M1 (Bepler et al., 1987). the multidrug resistant (MDR) SCLC cell lines used were NCI-H69/DAU4, NCI-H69/VP, and OC-NYH/VM selected for resistance to daunorubicin, VP-16, and VM-26 respectively. NCI-H69/DAU4 is a classical MDR cell line with P-glycoprotein in the cell membrane (Jensen et al., 1989b), OC-NYH/VM is resistant due to altered topoisomerase II activity (Jensen et al., 1991b), and NCI-H69/VP is resistant to vincristine as NCI-H69/DAU4, but is without P-glycoprotein expression and the mechanism(s) responsible for resistance in this line is still under investigation (Jensen et al., 1992). Resistant cell lines were grown in vitro without drug for a minimum of 5 days before testing. All cell lines were maintained at 37°C in RPMI 1640 with 10% foetal calf serum in a humidified atmosphere with 7.5% $CO_2$. At regular intervals the panel of cell lines was re-established from frozen sub-cultures to reduce or avoid sensitivity drifting. The cell lines were free of mycoplasm contamination DNA content (Vindeløv & Christensen, 1990), plating efficiency, relation to chemotherapy, and growth behaviour in vitro of the cell lines used are described in Table I. Also, Table I shows the relative resistance of H69/DAU4, H69/VP, and NYH/VM to doxorubicin (DOX), vincristine (VCR) and VP-16.

### Determination of $^3$H-daunorubicin accumulation

This was performed as described by Skovsgaard (1978) with the modification that the SCLC cells in single cell suspensions were incubated for 15 min with DNase I (0.025% w/v) (Sigma) before being exposed to daunorubicin (Versantvoort et al., 1992). This enzyme effectively dissolves the DNA in nuclei from dead cells whereby the background binding was significantly reduced (data not shown). Thereafter, single cell suspensions of $2.5 \times 10^6$ viable cells ml$^{-1}$ in 2 ml ($5 \times 10^6$ cells) were incubated with 3μM [$^3$H] daunorubicin for 60 min in standard phosphate buffer (57.0 mM NaCl, 5.0 mM KCl, 1.3 mM $MgSO_4$, 51.0 mM $Na_2HPO_4$, 9.0 mM $NaH_2PO_4$, pH 7.45) to which 5% (v/v) foetal calf serum was added. Incubations were performed with 10 mM glucose added, with 10 mM sodium azide ($NaN_3$) without glucose, with 10 mM sodium azide and 10 mM glucose, or with 10 mM glucose and 5μM verapamil. The cells were then spun down at 150g for 5 min and washed twice with 10 ml of ice-cold PBS. The cell pellets were solubilised in 0.8 ml 0.5 N KOH at 70°C for 1 h and analysed for $^3$H in a Packard scintillation spectrometer (Skovsgaard, 1978).

### Clonogenic assay

We have previously demonstrated that the comparison of effects of different drugs in a cell line is more reliable when the drugs are compared in simultaneous experiments on the same batch of cells (Jensen et al., 1989a). In our attempts to rationalize 'in vitro phase II trials in SCLC' the task was therefore to obtain more dose-response curves on one batch of cells. Since manual colony counting would be prohibitively laborious, we developed an automatic colony counter consisting of a videocamera built around a SB1024 image processing board (Bio-Rad Scan-Beam, Hadsund, Denmark) equipped with a motor steering for counting of 18 dishes. This enabled the automatic counting of colonies in 200 Petri dishes a day, with minimal expenditure of labour. In the experiments, single-cell suspensions ($1-4 \times 10^4$ cells ml$^{-1}$) in RPMI 1640 supplemented with 10% foetal calf serum were plated in soft agar on a feeder layer containing sheep red blood cells (Roed et al., 1987) in 35 mm petri dishes with the desired drug concentrations (continuous incubation). The number of cells were adjusted to obtain 2000–3000 colonies in the control dishes. In each experiment all 19 drugs (three concentrations of each, all plated in triplicate) and six control triplicates were tested on the same batch of cells. Solvent concentrations never exceeded 1% and had no influence on the plating efficiency. Plating was effectuated within one hour as the intraexperimental variation in plating efficiency of the controls exceeded 10% in more prolonged experiments. After 14–21 days the colonies were counted on the image analysis system. Colonies larger than 50μM in diameter were regarded

**Table I** DNA content, plating efficiency, relation to chemotherapy, and growth behavior *in vitro* and relative resistance of the cell lines used

| Cell line | DI | %PE | Prior therapy | Growth behaviour | DOX | RR VCR | VP-16 |
|---|---|---|---|---|---|---|---|
| NCI-H69 | 0.90 | 12 | Yes | S | | | |
| H69/DAU4 | 0.87 | 12 | | S | 5.1 | 5.1 | 2.3 |
| H69/VP | 0.82 | 13 | | S | 6.3 | 7.9 | 17 |
| OC-NYH | 1.39 | 27 | No | Mon | | | |
| NYH/VM | 1.29 | 30 | | Mon | 2.9 | 0.9 | 12 |
| NCI-N592 | 1.48 | 30 | Yes | S | | | |
| OC-TOL | 1.40 | 22 | No | S | | | |
| GLC-16 | 1.80 | 17 | Yes | S | | | |
| SCCL 86M1 | 1.45 | 15 | No | S | | | |

DI DNA index; PE plating efficiency at approximately 3000 colonies; S (growth in suspension) Mon (growth as monolayer); RR relative resistance i.e. the ratio of the LD50 value for the resistant over that for the wild-type parent line, data from Jensen et al. (1992).

as positive. The colony counter was interfaced with an IBM PS-2 60 computer and data was stored and analysed through use of SAS software. The dose reducing the number of colonies to 50% of control (LD50) was determined from three drug concentration points in linear regression analysis on logarithmically transformed response-data (Jensen *et al.*, 1989*a*). The drug concentrations chosen approximated to LD10, LD50, and LD90 obtained on cell line OC-NYH from dose-response curves in previous experiments and were as follows (μM): BCNU (2.3; 7.0; 14), 5FU (0.8; 1.2; 2.0), ACLA (0.0037; 0.012; 0.025), DOX (0.018; 0.037; 0.055), MEL (0.033; 0.1; 1), ARAC (0.025; 0.075; 0.15), BLEO (0.007; 0.07; 0.35), CAM (0.0006; 0.0022; 0.0036), CIS (0.33; 1.4; 3.3), HYD (39; 79; 158), IOD (0.001; 0.005; 0.010), MAMSA (0.01; 0.05; 0.1), MTX (0.02; 0.06; 0.11), MITO (0.007; 0.014; 0.045), MTC (0.009; 0.015; 0.021), VCR (0.0003; 0.001; 0.002), VDS (0.0005; 0.001; 0.002), VM26 (0.01; 0.02; 0.05), and VP16 (0.075; 0.125; 0.3). When the calculated LD50 values were above 3 × the highest tested concentration, the LD50 was assigned this value (i.e. 3 × LD90 on OC-NYH). Such 'horizontal' dose-response curves were only observed with some of the 'MDR' drugs in the resistant lines and accordingly their precise relative resistance is not defined. The relative resistance obtained in previous experiments with properly adjusted doses are shown in Table I. Patterns in sensitivity were studied by correlation analysis using rank orders of sensitivity with all possible pairings of the 19 agents. LD50 values from two experiments on each cell line were included in the analysis. Computations utilised correlation coefficients calculated as Spearman rank order correlations.

### Results

*Sensitivity pattern in three different MDR cell lines*

The cytotoxicity of the 19 anticancer agents was determined in a clonogenic assay as described in Materials and methods. The relative sensitivity to the 19 drugs in the 'wild type' cell line NCI-H69 compared to NCI-H69/DAU4 is shown in Figure 1a. In Figure 1b NCI-H69 is compared to NCI-H69/VP, and in Figure 1c the 'wild type' cell line OC-NYH is compared to OC-NYH/VM. The interexperimental variation on NCI-H69 is large (the coefficient of variation CV > 50%) with MTX, 5-FU and bleomycin whereas CV < 50% to the rest of the drugs and most often CV < 25%. Both resistant sublines of H69 exhibit cross-resistance to the vinca alkaloids VCR and VDS, to the anthracyclines doxorubicin (DOX) and aclarubicin (ACLA), the anthracenidone mitoxantrone (MIT), and to the 'classical' topoisomerase II targeting drugs VP-16, VM-26 and m-AMSA (MAM). Interestingly NCI-H69/VP appears sensitive to iododoxorubicin (IOD). Both resistant sublines of H69 exhibit collateral sensitivity to BCNU and Ara-C. Although not statistically significant both lines also seem collaterally sensitive to cisplatin (CIS), camptothecin (CAM), and hydroxyurea (HYD). In accordance to our previous data (Jensen *et al.*, 1989) the cell line NCI-H69 is four fold less sensitive to DOX than OC-NYH (LD50 0.091 and 0.023μM respectively). As the drug concentrations used were adjusted to OC-NYH sensitivity (please see materials and methods) this means that the estimated cross-resistance to DOX on H69/DAU4 is limited as compared to the data in Table I.

In contrast to the MDR sublines of H69, there is no cross-resistance to vinca alkaloids or to the anthracycline aclarubicin (ACLA) in the at-MDR cell line OC-NYH/VM. Compared to OC-NYH, the at-MDR cell line shows cross-resistance to doxorubicin, iododoxorubicin, mitoxantrone, m-AMSA and VP-16, and OC-NYH/VM also exhibits collateral sensitivity to hydroxyurea and Ara-C. There is no change in the sensitivity to camptothecin in this cell line. In Table II is summarised the LD50 values used in the computations of the relative sensitivities depicted in Figure 1. The median coefficient of variation (CV) for the uncertainty in the LD50 computations was 9% and the median CV for the repeated experiments was 30% (OC-NYH).

**Figure 1** The relative sensitivity to 19 anticancer agents in the multidrug resistant cell lines **a**, NCI-H69/DAU4 and **b**, NCI-H69/VP compared to the parental line NCI-H69 and in **c**, OC-NYH/VM compared to the parental line OC-NYH. In the figures are plotted the LD50 values obtained in three independent experiments on the parental lines (●) and two experiments on each of the resistant lines (★).

*Daunorubicin accumulation in the three different MDR cell lines*

We have previously shown that NCI-H69/DAU4 expresses P-glycoprotein (Jensen *et al.*, 1989b), whereas the protein is not detectable in the resistant sublines NCI-H69/VP and OC-NYH/VM (Jensen *et al.*, 1992). The cell line OC-NYH/VM has the characteristics of an at-MDR phenotype (Danks *et al.*, 1988), whereas NCI-H69/VP is cross-resistant to vincristine and the resistance mechanism(s) in this cell line is still unresolved. To further characterise the different resistant sublines of NCI-H69 and OC-NYH, the accumulation of [$^3$H] daunorubicin was determined. Daunorubicin is a well described P-glycoprotein substrate and as seen in Table III there is a reduced daunorubicin accumulation in H69/DAU4 as compared to the H69 'wild type'. When the H69/DAU4 cells are incubated without glucose and in the presence of azide,

**Table II** Mean LD50 values to 19 anticancer agents from three independent experiments in NCI-H69 and OC-NYH, and from two independent experiments in the MDR sublines H69/DAU4, H69/VP, and NYH/VM

| | | *Cell-Line* | | | | |
|---|---|---|---|---|---|---|
| *Drug* | | *NCI-H69* | *H69/DAU4* | *H69/VP* | *OC-NYH* | *NYH/VM* |
| 5FU | μM | 3.6 (2.4) | 2.5 (1.2) | 1.6 (0.06) | 2.2 (1.7) | 0.89 (0.14) |
| ACLA | nM | 12 (1.8) | 45 (4.5) | 17 | 8.2 (2.5) | 7.3 (0.2) |
| DOX | nM | 91 (15) | 134 (44) | 166 (★) | 23 (6) | 134 (44) |
| MEL | nM | 621 (165) | 846 (173) | 407 (112) | 431 (196) | 519 (39) |
| ARAC | nM | 149 (33) | 44 (16) | 85 (15) | 55 (5.6) | 34 (5.7) |
| BCNU | nM | 4.2 (0.9) | 2.3 (0.2) | 2.5 | 8.6 (5) | 4.9 (0.6) |
| BLEO | μM | 42 (31) | 28 (11) | 35 (20) | 80 (50) | 35 (0) |
| CAM | nM | 3.7 (0.5) | 2.5 (0.5) | 2.3 (1.5) | 1.6 (0.4) | 1.5 (0.2) |
| CIS | nM | 619 (160) | 443 (13) | 417 (72) | 726 (185) | 709 (36) |
| HYD | μM | 299 (65) | 192 (20) | 119 (1) | 83 (3) | 58 (7) |
| IOD | nM | 8.3 (1.5) | 19 (2.4) | 4.8 (2.6) | 2.4 (1) | 4.7 (1) |
| mAMSA | nM | 125 (29) | 300 (★) | 230 (99) | 80 (31) | 300 (★) |
| MTX | nM | 179 (144) | 330 (★) | 269 (86) | 28 (12) | 20 (1) |
| MIT | nM | 42 (2.7) | 127 (12) | 64 (2) | 10 (3) | 27 (2) |
| MTC | nM | 42 (15) | 58 (2) | 28 (7) | 19 (7) | 10 (3) |
| VCR | nM | 1.2 (0.2) | 6.0 (★) | 4.5 (0.9) | 0.8 (0.3) | 0.8 (0) |
| VDS | nM | 1.3 (0.2) | 7.6 (2) | 7.1 (2.4) | 1.4 (0.3) | 1.8 (0.8) |
| VM26 | nM | 57 (4) | 150 (★) | 150 (★) | 14 (1.6) | 150 (★) |
| VP16 | nM | 489 (94) | 900 (★) | 900 (★) | 100 (17) | 900 (★) |

Numbers in parenthesis are standard deviations. Repeated LD50 values to ACLA and BCNU are missing on cell line H69/VP.
★indicate that the LD50 value is 3 × higher than the highest dose tested.

**Table III** [$^3$H] Daunorubicin accumulation in SCLC cells pretreated with DNase I

| | *Accumulation in pmol daunorubicin/10$^6$ cells* | | | | | | | | |
|---|---|---|---|---|---|---|---|---|---|
| | GLU | SD | AZID | SD | GLAZ | SD | GLV5 | SD | N |
| NCI-H69 | 544 | (39) | 699 | (32) | 551 | (37) | 514 | (90) | 6 |
| H69/DAU4 | 170 | (55) | 488 | (51) | 197 | (61) | 333 | (37) | 5 |
| H69/VP | 324 | (57) | 719 | (13) | 496 | (34) | 588 | (10) | 5 |
| OC-NYH | 1078 | (77) | 1192 | (113) | 987 | (108) | 1035 | (87) | 5 |
| NYH/VM | 1077 | (128) | 1210 | (169) | 975 | (110) | 988 | (112) | 5 |
| | *Accumulation in % of the parent wild type line* | | | | | | | | |
| NCI-H69 | 100 | (7) | 128 | (6) | 101 | (7) | 94 | (15) | |
| H69/DAU4 | 31 | (10) | 90 | (9) | 36 | (11) | 61 | (7) | |
| H69/VP | 60 | (10) | 132 | (3) | 92 | (6) | 108 | (2) | |
| OC-NYH | 100 | (7) | 110 | (10) | 92 | (10) | 96 | (8) | |
| NYH/VM | 100 | (12) | 112 | (16) | 90 | (10) | 92 | (10) | |

SCLC cells were incubated for 60 min at 37°C with 3 μM daunorubicin in: GLU, medium containing 10 mM glucose; AZID, medium without glucose with 10 mM azid; GLAZ, medium with 10 mM glucose and 10 mM azid; GLV5, medium with 10 mM glucose and 5 μM verapamil. Hereafter the cells were washed at 4°C and drug accumulation was determined. Numbers in parenthesis are standard deviations.

there is a 3-fold increase in daunorubicin accumulation almost reaching a 'wild type' level. If energy production is restored as seen by the effect of addition of glucose to the azide incubations the drug accumulation is reduced. This effect is similar to the results obtained by Danø (1973) on the classic P-glycoprotein expressing MDR Ehrlich ascites tumour cells. As expected, verapamil which is a modulator of P-glycoprotein mediated resistance is also able to increase the drug accumulation in H69/DAU4. A similar pattern is observed in the P-glycoprotein negative H69/VP with a 2-fold increase in drug content when the cells are incubated with azide and a significant reduction by addition of glucose. Thus, in both MDR sublines of H69 one explanation of the resistance is a reduced drug accumulation. In contrast, there is no difference in daunorubicin accumulation in OC-NYH and OC-NYH/VM (Table III) although the latter is four fold less sensitive to daunorubicin (Jensen *et al.*, 1991*b*), confirming that this cell line fits the at-MDR phenotype (Danks *et al.*, 1988).

### *Sensitivity patterns in six wild type and three MDR lines*

The 19 drugs were tested on four additional 'wild type' cell lines and Figure 2 shows the cytotoxicities of the drugs against all six wild type cell lines and the three MDR SCLC cell lines as determined by clonogenic assay. The cell lines are sorted by increasing sensitivity to BCNU. Sensitivity variation across all lines to BCNU ranges from LD50 = 1.6μM on GLC-16 to LD50 = 14.0μM on cell line OC-TOL. The data demonstrates that the variation in sensitivity to most drugs in the 'wild type' lines is within a factor of 2–10. Sensitivity patterns to VP-16 and BCNU are very different, whereas patterns to VP-16 and VM-26 are identical and similar to the pattern obtained with doxorubicin. Similarity in sensitivity patterns are also obtained between vincristine and vindesine, as well as to BCNU and cisplatin.

However, a more detailed visual comparison of patterns of all possible drug pairings is obviously not feasible. We therefore performed a correlation analysis using rank orders of sensitivity with all possible pairings on the 19 agents. From such an analysis, a high correlation coefficient for a given pair of compounds is indicative of a similar pattern in response in the set of cell lines. A numerically low coefficient indicates that the two compounds are acting in different ways, and a negative correlation coefficient suggests that two drugs exhibit collateral sensitivity. Two separate experiments from each of the nine SCLC cell lines (i.e. 18 LD50 values for each drug) were used in the correlation analysis. Table IV-A presents the correlation coefficients to all the possible pairings. In accordance with the visual interpretation of the data depicted in Figure 2, BCNU and cisplatin, VP-16 and VM-26, as well as vincristine and vindesine have very high

**Figure 2** Sensitivity patterns to 19 anticancer agents on nine SCLC cell lines. The results are depicted as the mean LD50 values from at least two experiments and the cell lines are sorted by increasing sensitivity to BCNU. The three MDR lines are demarcated with solid bars.

correlation coefficients (89%, 99%, and 89% respectively). The sensitivity pattern to VP-16 is highly correlated to the pattern to doxorubicin (92%), and the drug also exhibits high correlation coefficients to the other topoisomerase II targeting agents mitoxantrone (81%) and m-AMSA (72%). Table IV-A depicts a remarkable inverse correlation between BCNU and MTX (−66%) as well as between bleomycin and hydroxyurea (−61%). In addition, numerically smaller inverse correlation coefficients are observed a number of times. Thus the alkylating agents (BCNU and cisplatin), the antimetabolites as well as bleomycin are all inversely correlated to the 'MDR' drugs, e.g. BCNU and bleomycin correlation to mitoxantrone is −45% and −55% respectively. Table IV-B shows correlation coefficients on the six wild type lines alone, and thereby the consequence of including the MDR lines in the cell line panel can be seen by comparing table IV-A to IV-B. Although VP-16 and the vinca alkaloids have different mechanisms of action, there is a clear correlation between sensitivity patterns to these two drug types not only in the full panel of nine SCLC lines

**Table IV** A) Correlation analysis on rank order of sensitivity with all possible pairings of the 19 anticancer agents. Correlation coefficients (%) were obtained in six 'wild type' and three MDR lines

| | *BCNU* | *5FU* | *ACLA* | *DOX* | *MEL* | *ARAC* | *BLEO* | *CAM* | *CIS* | *HYD* | *IOD* | *mAMSA* | *MTX* | *MITO* | *MTC* | *VCR* | *VDS* | *VM26* | *VP16* |
|---|---|---|---|---|---|---|---|---|---|---|---|---|---|---|---|---|---|---|---|
| BCNU | ★ | −26 | −37 | −13 | −6 | 2 | 40 | −35 | 89 | −47 | −31 | −44 | −66 | −45 | −25 | −19 | −10 | −19 | −21 |
| 5FU | −26 | ★ | 22 | −47 | 76 | 43 | 29 | 24 | −23 | 6 | 28 | −7 | 44 | −33 | 24 | −4 | −4 | −38 | −34 |
| ACLA | −37 | 22 | ★ | 59 | 37 | −8 | 7 | 45 | −21 | 14 | 76 | 27 | 56 | 60 | 48 | 67 | 60 | 50 | 47 |
| DOX | −13 | −47 | 59 | ★ | −12 | −18 | −32 | 18 | −2 | 20 | 43 | 56 | 22 | 84 | 30 | 67 | 69 | 94 | 92 |
| MEL | −6 | 76 | 37 | −12 | ★ | −1 | 36 | 34 | 12 | −20 | 52 | 29 | 13 | −17 | 13 | 15 | 16 | −0 | 5 |
| ARAC | 2 | 43 | −8 | −18 | −1 | ★ | 0 | 31 | −12 | 54 | 0 | −23 | 30 | −23 | 49 | 1 | −2 | −13 | −15 |
| BLEO | 40 | 29 | 7 | −32 | 36 | 0 | ★ | −2 | 34 | −61 | −19 | −14 | −22 | −55 | −22 | −22 | −6 | −42 | −43 |
| CAM | −35 | 24 | 45 | 18 | 34 | 31 | −2 | ★ | −12 | 47 | 73 | 44 | −2 | 23 | 13 | 44 | 34 | 16 | 14 |
| CIS | 89 | −23 | −21 | −2 | 12 | −12 | 34 | −12 | ★ | −47 | −9 | −22 | −59 | −34 | −23 | −18 | −13 | −7 | −6 |
| HYD | −47 | 6 | 14 | 20 | −20 | 54 | −61 | 47 | −47 | ★ | 42 | 15 | 44 | 50 | 66 | 52 | 30 | 32 | 31 |
| IOD | −31 | 28 | 76 | 43 | 52 | 0 | −19 | 73 | −9 | 42 | ★ | 47 | 27 | 56 | 43 | 63 | 54 | 48 | 48 |
| mAMSA | −44 | −7 | 27 | 55 | 29 | −23 | −14 | 44 | −22 | 15 | 47 | ★ | 18 | 50 | 0 | 56 | 59 | 71 | 72 |
| MTX | −66 | 44 | 56 | 22 | 13 | 30 | −22 | −2 | −59 | 44 | 27 | 18 | ★ | 49 | 73 | 41 | 41 | 29 | 29 |
| MITO | −45 | −33 | 60 | 84 | −17 | −23 | −55 | 23 | −34 | 50 | 56 | 50 | 49 | ★ | 48 | 76 | 62 | 83 | 81 |
| MTC | −25 | 24 | 48 | 30 | 13 | 50 | −22 | 13 | −23 | 66 | 43 | 0 | 73 | 48 | ★ | 55 | 41 | 36 | 37 |
| VCR | −19 | −4 | 67 | 67 | 15 | 1 | −22 | 44 | −18 | 52 | 63 | 56 | 41 | 76 | 55 | ★ | 89 | 72 | 73 |
| VDS | −10 | −4 | 60 | 69 | 16 | −2 | −6 | 34 | −13 | 30 | 54 | 59 | 41 | 62 | 41 | 89 | ★ | 71 | 71 |
| VM26 | −19 | −38 | 50 | 94 | 0 | −13 | −42 | 16 | −7 | 32 | 48 | 71 | 29 | 83 | 36 | 72 | 71 | ★ | 99 |
| VP16 | −21 | −34 | 47 | 92 | 5 | −15 | −43 | 14 | −6 | 31 | 48 | 72 | 29 | 81 | 37 | 73 | 71 | 99 | ★ |

B) Correlation coefficients (%) in six 'wild type' lines

| | *BCNU* | *5FU* | *ACLA* | *DOX* | *MEL* | *ARAC* | *BLEO* | *CAM* | *CIS* | *HYD* | *IOD* | *mAMSA* | *MTX* | *MITO* | *MTC* | *VCR* | *VDS* | *VM26* | *VP16* |
|---|---|---|---|---|---|---|---|---|---|---|---|---|---|---|---|---|---|---|---|
| ARAC | −10 | 41 | 2 | 13 | 6 | ★ | −22 | 42 | −10 | 57 | 23 | 8 | 55 | −2 | 77 | 26 | 32 | 42 | 36 |
| BCNU | ★ | −12 | 1 | −9 | 22 | −10 | 47 | −40 | 93 | −32 | 5 | −50 | −48 | −28 | 25 | 42 | 26 | −13 | −16 |
| VCR | 42 | −27 | 29 | 53 | 12 | 26 | 1 | 82 | 55 | 34 | 63 | 35 | −54 | 51 | 17 | ★ | 88 | 67 | 68 |
| VP16 | −16 | −7 | 44 | 84 | 20 | 36 | −44 | 82 | 6 | 50 | 92 | 51 | 0 | 71 | 30 | 68 | 57 | 97 | ★ |

**Figure 3** Correlation coefficients between rank orders in sensitivity to 19 anticancer agents in nine SCLC cell lines and the epipodophyllotoxin VP-16 are shown on the abscissa. The ordinate depicts correlation coefficients between the 19 agents and the alkylating agent BCNU. Different drug types are demarcated in plot.

(Table IV-A) but also in the six 'wild types' (Table IV-B). In contrast, both Ara-C and camptothecin demonstrate notable reduced correlation coefficients to VP-16 when the MDR lines are included. This reflects the collateral sensitivity exhibited by these compounds in the MDR lines (Figure 1).

In order to visualise some of the data obtained on the full cell line panel (Table IV-A), the correlations of the 19 drug-patterns to the VP-16 sensitivity pattern were plotted against the corresponding coefficients to BCNU (Figure 3). This plot exhibits the remarkable feature of drug-type specific clustering. The topoisomerase II targeting drugs are clustered together, as are the vinca alkaloids, and the lipophilic anthracyclines (iododoxorubicin and aclarubicin). Although more disparate, there also seem to be areas occupied by alkylating agents and pyrimidine antimetabolites (ara-C and 5-FU). In addition, this plot demonstrates that drugs as ara-C and camptothecin exhibit sensitivity patterns that differ both from VP-16 and from the alkylating agents BCNU and cisplatin. Thereby, the differential sensitivity patterns obtained in the cell line panel enable the selection of non-cross-resistant drugs.

### Discussion

The present study demonstrates that the clonogenic assay used on this panel of SCLC cell lines is capable of displaying both cell-determined and structure-determined differences in the cytotoxicity of the drugs. As such the panel may provide a useful adjunct to other tumour models in the selection of non-cross-resistant anticancer agents and in elucidating mechanisms of action of new drugs. That such patterns in sensitivity may give clues to the mechanism of action was recently described by Bai *et al.* (1991). Thus, in the National Cancer Institute (NCI, Bethesda, USA) drug evaluation program the two marine natural products halichondrin B and homohalichondin B exhibited sensitivity patterns similar to the antimitotic agent vincristine and it was subsequently demonstrated that these potential new drugs are antimitotic agents which interact with tubulin.

We chose to include experimentally developed resistant cell lines in our panel. Such cell lines can be valuable when displaying structurally determined differences in the cytotoxicity of drugs. We have previously shown that the anthracycline aclarubicin (ACLA) does not stimulate topoisomerase II mediated DNA breaks (Jensen *et al.*, 1991*a*). This is in contrast to other intercalating agents as doxorubicin and mitoxantrone. The fact that there is cross-resistance to these two drugs but not to ACLA in the at-MDR line OC-NYH/VM (Figure 1c) strongly supports the idea that the cytotoxicity of ACLA, in contrast to doxorubicin and mitoxantrone, is independent of topoisomerase II activity (Jensen *et al.*, 1991*b*). While experimentally developed resistant cell lines may elucidate drug mechanism of action it is debated whether such resistant cell lines reflect a relevant clinical heterogeneity. An argument against the experimental lines is that the high degree of resistance often seen in these lines is far above what seems clinically relevant. As maximum tolerated doses are often used, a 2–3 × decrease in sensitivity is sufficient to explain clinical resistance. However, recent clinical investigations demonstrate that MDR is not only a laboratory investigation (Nooter & Herweijer, 1991). In patients with acute myelocytic leukaemia there is a significantly reduced survival of patients with overexpression of *mdrl* mRNA in the leukaemia cells (Pirker *et al.*, 1991). It is still not settled whether the classical P-glycoprotein MDR phenotype is involved in drug resistance in SCLC. P-glycoprotein positive SCLC cell lines have been generated in the laboratory by us and others (Reeve *et al.*, 1989; Minato *et al.*, 1990; Jensen *et al.*, 1989*b*), but the protein appears to be rarely expressed in SCLC tumor biopsies (Nooter & Heerweijer, 1991). Thus, other mechanisms of resistance may be relevant in SCLC. The presence of alternative transport-proteins have been suggested by Versantvoort *et al.* (1992) and Cole *et al.* (1991) in their experimental MDR SCLC cell lines, and our data on the P-glycoprotein negative H69/VP cell line also suggest a MDR mechanism involving an active drug efflux. Also alterations in topoisomerase II have been described in experimentally resistant SCLC lines (de Jong *et al.*, 1990; Jensen *et al.*, 1991b; Cole *et al.*, 1991). The lack of data on topoisomerase II activity in patient biopsies still only enables speculations on its clinical relevance. Therefore, we chose to include different MDR phenotypes in our cell line panel in an attempt to cover the whole (known) spectrum of VP-16 resistance mechanisms.

The clonogenic assay is laborious and accordingly a number of alternative, easier assys have been developed for large-scale screening, e.g. MTT and protein assays (Carmichael *et al.*, 1988; Rubinstein *et al.*, 1990). Instead of using one of these methods we have rationalised the techniques in the clonogenic assay. Cell handling and interexperimental variation was reduced by using continuous drug exposure (Jensen *et al.*, 1989*a,b*), drugs were frozen in 'ready to use' concentrations, and the time consuming colony counting was automatized. Thereby, it became possible to obtain dose response curves to 19 drugs simultaneously in a cell line. Campling *et al.* (1991), Tasi *et al.* (1990), Smit *et al.* (1992), and Carmichael *et al.* (1988) have recently published results from MTT sensitivity testing in panels of SCLC cell lines. In accordance with our findings, Campling *et al.* (1991) found trends of collateral sensitivity to cisplatin, carboplatin, and nitrogen mustard in a MDR subline of NCI-H69 selected for resistance to doxorubicin. However, Tsai *et al.* (1990) did not observe a differential cytotoxicity pattern to VP-16 and cisplatin in 27 'wild type' SCLC cell lines. In contrast to our observations, cell lines resistant to the one drug was resistant to the other and vice versa. More in agreement with our observations, Carmichael *et al.* (1988) found lack of correlation between VP-16 and cisplatin sensitivity in 15 'wild type' SCLC cell lines. There are several explanations to this variance in sensitivity patterns. Thus, different cell lines and assays were employed. The MTT assay measures a combination of drug-induced cytotoxicity and inhibition of cell growth (Campling *et al.*, 1991), whereas the clonogenic assay measures the loss of reproductive potential in single cells. More importantly, however, the end point of the different investigations differ. Campling *et al.* (1991), Smit *et al.* (1992), and Tsai *et al.* (1990) correlated *in vitro* sensitivity data with clinical outcome, whereas our focus is the comparison of drugs. Our attempt has been to select and develop a cell line panel as a model of SCLC tumours reflecting differential sensitivity patterns. Until now, we have concentrated our efforts on the various VP-16 resistant phenotypes. In the clinic, drug resistance is not confined to VP-16 and an ideal cell line panel should probably include cell lines with resistance to all important drug types. Fortunately it appears that our panel does include cell lines with an intrinsic resistance towards alkylating agents e.g. OC-TOL and OC-NYH. In addition, we are in the process of developing cell lines with resistance to both VP-16 and cisplatin.

The value of a drug screening model is ultimately established by its ability to identify combinations of drugs or new compounds which are useful in clinical treatment. In this context it is important that the model reflects experimental and clinical experience. We feel that our model is supported by the following findings:

(1) The clinical synergy between cisplatin and VP-16 in SCLC (Porter *et al.*, 1985) seems to be reflected in our panel by the lack of cross-resistance or even collateral sensitivity between cisplatin and VP-16 (e.g. in H69/DAU4).

(2) Similarly, our data demonstrate a lack of cross-resistance between topoisomerase II targeting agents e.g. mitoxantrone and the antimetabolite ara-C. This combination is widely used in the treatment of acute myelocytic leukaemia.

(3) We find very high correlations between the two epipodophyllotoxins VP-16 and VM-26 supporting the clinical evidence that these drugs are similar (Bork *et al.*, 1991).

(4) It is debated whether topoisomerase II is the primary target of doxorubicin and in this context it is interesting that the two 'classic' topoisomerase II targeting agents VP-16 and VM-26 also exhibit a very high correlation to doxorubicin. Similar results on a large panel of 62 various human tumor cell lines have recently been described from the NCI drug discovery program by Wu *et al.* (1992), with a correlation coefficient of 0.88 between VP-16 and doxorubicin.

Obviously, the treatment of SCLC with combinations of topoisomerase II targeting drugs and alkylating agents is not 'enough' to cure the patients (Cantwell *et al.*, 1988) and new drugs with activity in the doubly resistant cell populations are needed. The sensitivity correlations in Figure 3 and in Table IV-A show that sensitivity patterns to ara-C and camptothecin differ both from topoisomerase II targeting drugs and from alkylating agents, wherefore ara-C and camptothecin appear promising. While ara-C is not clinically active in SCLC, its analogue gemcitabin has demonstrated activity in non-small cell lung cancer (Anderson *et al.*, 1991). If gemcitabin exhibits a sensitivity pattern identical to ara-C, our data suggests that it could be useful in combination with alkylating agents and 'MDR drugs'. Drugs with effect on the 'new target' topoisomerase I also seem promising. There is no cross-resistance to camptothecin in MDR, indeed, two of our three MDR lines are collaterally sensitive to camptothecin. It is tempting to relate the inverse pattern of sensitivity to the fact that topoisomerase II is able to replace topoisomerase I *in vitro* (Liu *et al.*, 1989; Yang *et al.*, 1987). Cells that are resistant to camptothecin appear to depend to a greater extent than wild-type cells upon topoisomerase II activity. This in turn can lead to collateral sensitivity to topoisomerase II targeting agents (Sugimoto *et al.*, 1990). Such a hypersensitivity was recently described by Mattern *et al.* (1991), and we found a similar sensitivity pattern in the human RPMI8402/k5 cell line with an altered topoisomerase I (Kjeldsen *et al.*, 1988; Kjeldsen *et al.*, 1991). Thus, cells resistant to topoisomerase I inhibitors are, in some cases at least, hypersensitive to certain topoisomerase II inhibitors.

In conclusion the present study shows that it is feasible to include 19 drugs in simultaneous experiments in a clonogenic assay. The analysis of the differential cytotoxicity patterns in the panel of cell lines makes it possible (i) to obtain information about drug mechanism of action and enable combinations of non-cross-resistant drugs and (ii) to show where new drugs 'fit in' among established drugs. At present analogues of ara-C and topoisomerase I active drugs appear to be promising as they demonstrate low or negative correlation coefficients both to topoisomerase II targeting agents and to alkylating agents.

Annette Nielsen and Eva Høj are thanked for excellent technical assistance, and John Post for preparing the illustrations. Supported by grants from the Danish Cancer Society, the Danish Medical Research Council, and the Lundbeck Foundation.

### References

ANDERSON, H., THATCHER, N., HANSEN, H.H., LUND, B., WALLING, J. & HATTY, S. (1991). Gemcitabine (2'2'-difluorodeoxycytidine) in NSCLC-A phase II study. *Eur. J. Cancer*, **supp. 2**, 196.

BAI, R., PAULL, K.D., HERALD, C.L., MALSPEIS, L., PETTIT, G.R. & HAMEL, E. (1991). Halichondrin B and homohalichondrin B, marine natural products binding in the vinca domain of tubulin. Discovery of tubulin-based mechanism of action by analysis of differential cytotoxicity data. *J. Biol. Chem.*, **266**, 15882-15889.

BERENDSEN, H.H. DE LEIJ, L., DE VRIES, E.G.E., MESANDER, G., MULDER, N.H., DE JONG, B., BUYS, C.H.C.M., POSTMUS, P.E., POPPEMA, S., SLUITER, H.J. & THE, H.T. (1988). Characterization of Three Small Cell Lung Cancer Cell Lines Established from One Patient during Longitudinal Follup-up. *Cancer Res.*, **48**, 6891-6899.

BEPLER, G., JAQUES, G., NEUMANN, K., AUMULLER, G., GROOP, C. & HAVEMANN, K. (1987). Establishment, growth properties, and morphological characteristics of permanent human small cell lung cancer cell lines. *J. Cancer Res. Clin. Oncol.*, **113**, 31–40.

BORK, E., ERSBØLL, J., DOMBERNOWSKY, P., BERGMAN, B., HANSEN, M. & HANSEN, H.H. (1991). H. Teniposide and etoposide in previously untreated small cell lung cancer. A randomized study. *J. Clin. Oncol.*, **9**, 1627–1631.

CAMPLING, B.G., PYM, J., BAKER, H.M., COLE, S.P.C. & LAM, Y.M. (1991). Chemosensitivity testing of small cell lung cancer using the MTT assay. *Br. J. Cancer*, **63**, 75–83.

CANTWELL, B.M.J., BOZZINO, J.M., CORRIS, P. & HARRIS, A.L. (1988). The Multidrug Resistant Phenotype in Clinical Practice; Evaluation of Cross Resistance to Ifosfamide and Mesna after VP16-213, Doxorubicin and Vincristine (VPAV) for Small Cell Lung Cancer. *Eur. J. Cancer Clin. Oncol.*, **24**, 123–129.

CARMICHAEL, J., MITCHELL, J.B., DEGRAFF, W.G., GAMSON, J., GAZDAR, A.F., JOHNSON, B.E., GLATSTEIN, E. & MINNA, J.D. (1988). Chemosensitivity testing of human lung cancer cell lines using the MTT assay. *Br. J. Cancer*, **57**, 540–547.

CARNEY, D.N., GAZDAR, A.F., BEPLER, G., GUCCION, J.G., MARANGOS, P.J., MOODY, T.W., ZWEIG, M.H. & MINNA, J.D. (1985). Establishment and identification of small cell lung cancer cell lines having classic and variant features. *Cancer Res.*, **45**, 2913–2923.

COLE, S.P.C., (1992) Multidrug resistance in small cell lung cancer. *Can. J. Physiol. Pharmacol.*, **70**, 313–329.

COLE, S.P.C., CHANDA, E.R., DICKE, F.P., GERLACH, J.H. & MIRSKI, S.E.L. (1991). Non-P-glycoprotein-mediated multidrug resistance in a small cell lung cancer cell line: Evidence for decreased susceptibility to drug-induced DNA damage and reduced levels of topoisomerase II. *Cancer Res.*, **51**, 3345–3352.

COLEY, H.M., WORKMAN, P. & TWENTYMAN, P.R. (1991). Retention of activity by selected anthracyclines in a multidrug resistant human large cell lung carcinoma line without P-glycoprotein hyperexpression. *Br. J. Cancer*, **63**, 351–357.

DANKS, M.K., SCHMIDT, C.A., CIRTAIN, M.C., SUTTLE, D.P. & BECK, W.T. (1988). Altered Catalytic Activity of and DNA Cleavage by DNA Topoisomerase II from Human Leukemic Cells Selected for Resistance to VM-26. *Biochemistry*, **27**, 8861–8869.

DANØ, K. (1973). Active outward transport of daunomycin in resistant Ehrlich ascites tumor cells. *Biochim. Biophys. Acta*, **323**, 466–483.

DE JONG, S., ZIJLSTRA, J.G., DE VRIES, E.G.E. & MULDER, N.H. (1990). Reduced DNA Topoisomerase II Activity and Drug-induced DNA Cleavage Activity in an Adriamycin-resistant Human Small Cell Lung Carcinoma Cell Line. *Cancer Res.*, **50**, 304–309.

DE LEIJ, L., POSTMUS, P.E., BUYS, C.H.C.M., ELEMA, J.D., RAMAEKERS, F., POPPEMA, S., BROUWER, M., VAN DER VEEN, A.Y., MESANDER, G. & THE, T.H. (1985). Characterization of three new variant type cell lines derived from small cell carcinoma of the lung. *Cancer Res.*, **45**, 6024–6033.

DE VRIES, E.G.E., MEIJER, C., TIMMER-BOSSCHA, H., BERENDSEN, H.H., DE LEIJ, L., SCHEPER, R.J. & MULDER, N.H. (1989). Resistance Mechanisms in Three Human Small Lung Cancer Cell Lines Established from One Patient during Clinical Follow-up. *Cancer Res.*, **49**, 4175–4179.

ENDICOTT, J.A. & LING, V. (1989). The biochemistry of P-glycoprotein-mediated multidrug resistance, *Ann. Rev. Biochem.*, **58**, 137–171.

FERGUSON, P.J., FISHER, M.H., STEPHENSON, J., LI, D., ZHOU, B., CHENG, Y. (1988). Combined Modalities of Resistance in Etoposide-resistant Human KB Cell Lines. *Cancer Res.*, **48**, 5956–5964.

FRANCO, R., KRAFT, T., MILLER, T., POPP, M. & MARTELO, O. (1984). Storage of chemotherapy drugs for use in the human tumor stem cell assay. *Int. J. Cell Clon.*, **2**, 2–8.

HANSEN, H.H. (1992). Management of small-cell cancer of the lung. *The Lancet*, **339**, 846–849.

JENSEN, P.B., JENSEN, P.S., DEMANT, E.J.F., FRICHE, E., SØRENSEN, B.S., SEHESTED, M., WASSERMANN, K., VINDELØV, L., WESTERGAARD, O & HANSEN, H.H. (1991*a*). Antagonistic effect of aclarubicin on daunorubicin induced cytotoxicity in human small cell lung cancer cells: relationship to DNA integrity and topoisomerase II. *Cancer Res.*, **51**, 5093–5099.

JENSEN, P.B., JENSEN, P.S., SEHESTED, M., DEMANT, E.J.F., SØRENSEN, B.S., VINDELØV, L. & HANSEN, H.H. (1991*b*). Lack of cross-resistance to aclarubicin in an altered topoisomerase II multidrug resistant (at-MDR) small cell lung cancer (SCLC) cell line. *Proc. Am. Assoc. Cancer Res.*, **32**, 350.

JENSEN, P.B., ROED, H., SEHESTED, M., DEMANT, E.J.F., VINDELØV, L., CHRISTENSEN, I.J. & HANSEN, H.H. (1992). Doxorubicin sensitivity pattern in a panel of small cell lung cancer cell lines: Correlation to etoposide and vincristine and inverse correlation to carmustine sensitivity. *Cancer Chemother. Pharmacol.*, **31**, 46–52.

JENSEN, P.B., ROED, H., VINDELØV, L., CHRISTENSEN, I.J. & HANSEN, H.H. (1989*a*). Reduced variation in the clonogenic assay obtained by standardization of the cell culture conditions prior to drug testing on human small cell lung cancer cell lines. *Invest. New Drugs*, **7**, 307–315.

JENSEN, P.B., VINDELØV, L., ROED, H., DEMANT, E.J.F., SEHESTED, M., SKOVSGAARD, T. & HANSEN, H.H. (1989*b*). *In vitro* evaluation of the potential of aclarubicin in the treatment of small cell carcinoma of the lung (SCCL). *Br. J. Cancer*, **60**, 838–844.

KJELDSEN, E., BONVEN, B.J., ANDOH, T., ISHII, K., OKADA, K., BOLUND, L. & WESTERGAARD, O. (1988). Characterization of a Camptothecin-resistant Human DNA Topoisomerase I. *J. Biol. Chem.*, **263**, 3912–3916.

KJELDSEN, E., JENSEN, P.B., ALSNER, J., ANDOH, T., BOLUND, L. & WESTERGAARD, O. (1991). Altered DNA topoisomerase II expression in a campthothecin-resistant human lymphoblastoid cell line. International Symposium on DNA Topoisomerases in cancer chemotherapy, (abstract) Nagoya, Japan.

KRISTJANSEN, P.E.G. & HIRSCH, F.R. (1989). A review of the 5th world congress on lung cancer held by the International Association for the Study of Lung Cancer. *Eur. Respir. J.*, **2**, 275–279.

LIU, L.F. (1989). DNA topoisomerase poisons as antitumor drugs. *Annu. Rev. Biochem.*, **58**, 351–375.

LONG, B.H., WANG, L., LORICO, A., WANG, R.C.C., BRATTAIN, M.G. & CASAZZA, A.M. (1991). Mechanisms of resistance to etoposide and teniposide in acquired resistant human colon and lung carcinoma cell lines. *Cancer Res.*, **51**, 5275–5284.

MATTERN, M.R., HOFMANN, G.A., MCCABE, F.L. & JOHNSON, R.K. (1991). Synergistic cell killing by ionizing radiation and topoisomerase I inhibitor topothecan (SK&F 104864). *Cancer Res.*, **51**, 5813–5816.

MINATO, K., KANZAWA, F., NISHIO, K., NAKAGWA, K., FUJIWARA, Y. & SAIJO, N. (1990). Characterization of an etoposide-resistant human small-cell lung cancer cell line. *Cancer Chemother. Pharmacol.*, **26**, 313–317.

NOOTER, K. & HERWEIJER, H. (1991). Multidrug resistance (mdr) genes in human cancer. *Br. J. Cancer*, **63**, 663–669.

PIRKER, R., WALLNER, J., GEISSLER, K., LINKESCH, W., HAAS, O.A., BETTELHEIM, P., HOPFNER, M., SCHERRER, R., VALENT, P., HAVELEC, L., LUDWIG, H. & LECHNER, K. (1991). MDR1 gene expression and treatment outcome in acute myeloid leukemia. *J. Natl. Cancer Inst.*, **83**, 708–712.

PORTER, L.L., JOHNSON, D.H., HAINSWORT, J.D., HANDE, K.R. & GRECO, F.A. (1985). Cisplatin and etoposide combination chemotherapy for refractory small cell carcinoma of the lung. *Cancer Treat. Rep.*, **69**, 479–481.

REEVE, J.G., RABBITTS, P.H. & TWENTYMAN, P.R. (1989). Amplification and expression of mdrl gene in a multidrug resistant variant of small cell lung cancer line NCI-H69. *Br. J. Cancer*, **60**, 339–342.

ROED, H., CHRISTENSEN, I.J., VINDELØV, L., SPANG-THOMSEN, M. & HANSEN, H.H. (1987). Inter-experiment variation and dependence on culture conditions in assaying chemosensitivity of human small cell lung cancer lines. *Eur. J. Cancer Clin. Oncol.*, **23**, 177–186.

RUBINSTEIN, L.V., SHOEMAKER, R.H., PAULL, K.D., SIMON, R.M., TOSINI, S., SKEHAN, P., SCUDIERO, D.A., MONKS, A. & BOYD, M.R. (1990). Comparison of in vitro anticancer-drug-screening data generated with a tetrazolium assay versus a protein assay against a diverse panel of human tumor cell lines. *J. Natl. Cancer Inst.*, **82**, 1113–1118.

SEHESTED, M., FRICHE, E., JENSEN, P.B. & DEMANT, E.J.F. (1992). Relationship of VP-16 to the classical multidrug resistance (MDR) phenotype. *Cancer Res.*, **52**, 2874–2879.

SKOVSGAARD, T. (1978). Mechanisms of resistance to daunorubicin in Ehrlich ascites tumor cells. *Cancer Res.*, **38**, 1785–1791.

SMIT, E.F., DEVRIES, E.G.E., TIMMER-BOSSCHA, H., DELEIJ, L.F.H.M., OOSTERHUIS, J.W., SCHEPER, R.J., WEENING, J.J., POSTMUS, P.E. MULDER, N.H. (1992). *In vitro* response of human small-cell lung-cancer cell lines to chemotherapeutic drugs; no correlation with clinical data. *Int. J. Cancer*, **51**, 72–78.

SUGIMOTO, Y., TSUKAHARA, S., OH-HARA. T., LIU, L.F. & TSURUO, T. (1990). Elevated expression of DNA topoisomerase II in camptothecin-resistant human tumor cell lines. *Cancer Res.*, **50**, 7962–7965.

TSAI, C., IHDE, D.C., KKADYAMA, C., VENZON, D. & GAZDAR, A.F. (1990). Correlation of *in vitro* drug sensitivity testing of long-term small cell lung cancer lines with response and survival. *Eur. J. Cancer*, **26**, 1148-1152.

VERSANTVOORT, C.H.M., BROXTERMAN, H.J., PINEDO, H.M., DE VRIES, E.G.E., FELLER, N., KUIPER, C.M. & LANKELMA, J. (1992). Energy-dependent processes involved in reduced drug accumulation in multidrug-resistant lung cancer cell lines without P-glycoprotein expression. *Cancer Res.*, **52**, 17-23.

VINDELØV, L. & CHRISTENSEN, I.J. (1990). A review of techniques and results obtained in one laboratory by an integrated system of methods designed for routine flow cytometric DNA analysis. *Cytometry*, **11**, 753-770.

WU, L., SMYTHE, A.M., STINSON, S.F., MULLENDORE, L.A., MONKS, A., SCUDIERO, D.A., PAULL, K.D., KOUTSOUKOS, A.D., RUBINSTEIN, L.V., BOYD, M.R. & SHOEMAKER, R.H. (1992). Multidrug-Resistant Phenotype of Disease-Oriented Panels of Human Tumor Cell Lines Used for Anticancer Drug Screening. *Cancer Res.*, **52**, 3029-3034.

YANG, L., WOLD, M.S., LI, J.J., KELLY, T.J. & LIU, L.F. (1987). Roles of DNA topisomerases in simian virus 40 DNA replication *in vitro*. *Proc. Natl Acad. Sci.*, **84**, 950-954.